# Pre-Infection Innate Immunity Attenuates SARS-CoV-2 Infection and Viral Load in iPSC-Derived Alveolar Epithelial Type 2 Cells

**DOI:** 10.3390/cells13050369

**Published:** 2024-02-21

**Authors:** Satish Kumar, Jose Granados, Miriam Aceves, Juan Peralta, Ana C. Leandro, John Thomas, Sarah Williams-Blangero, Joanne E. Curran, John Blangero

**Affiliations:** 1 Division of Human Genetics and South Texas Diabetes and Obesity Institute, University of Texas Rio Grande Valley School of Medicine, McAllen, TX 78504, USA; jose.granados04@utrgv.edu (J.G.); miriam.aceves@utrgv.edu (M.A.); john.thomas@utrgv.edu (J.T.); 2 Division of Human Genetics and South Texas Diabetes and Obesity Institute, University of Texas Rio Grande Valley School of Medicine, Brownsville, TX 78520, USA; juan.peralta@utrgv.edu (J.P.); ana.leandro@utrgv.edu (A.C.L.); sarah.williams-blangero@utrgv.edu (S.W.-B.); joanne.curran@utrgv.edu (J.E.C.); john.blangero@utrgv.edu (J.B.)

**Keywords:** iPSCs, AT2s, COVID-19, in vitro modeling, innate immunity

## Abstract

A large portion of the heterogeneity in coronavirus disease 2019 (COVID-19) susceptibility and severity of illness (SOI) remains poorly understood. Recent evidence suggests that SARS-CoV-2 infection-associated damage to alveolar epithelial type 2 cells (AT2s) in the distal lung may directly contribute to disease severity and poor prognosis in COVID-19 patients. Our in vitro modeling of SARS-CoV-2 infection in induced pluripotent stem cell (iPSC)-derived AT2s from 10 different individuals showed interindividual variability in infection susceptibility and the postinfection cellular viral load. To understand the underlying mechanism of the AT2′s capacity to regulate SARS-CoV-2 infection and cellular viral load, a genome-wide differential gene expression analysis between the mock and SARS-CoV-2 infection-challenged AT2s was performed. The 1393 genes, which were significantly (one-way ANOVA FDR-corrected *p* ≤ 0.05; FC *abs* ≥ 2.0) differentially expressed (DE), suggest significant upregulation of viral infection-related cellular innate immune response pathways (*p*-value ≤ 0.05; activation *z*-score ≥ 3.5), and significant downregulation of the cholesterol- and xenobiotic-related metabolic pathways (*p*-value ≤ 0.05; activation *z*-score ≤ −3.5). Whilst the effect of post-SARS-CoV-2 infection response on the infection susceptibility and postinfection viral load in AT2s is not clear, interestingly, pre-infection (mock-challenged) expression of 238 DE genes showed a high correlation with the postinfection SARS-CoV-2 viral load (FDR-corrected *p*-value ≤ 0.05 and *r*^2^-absolute ≥ 0.57). The 85 genes whose expression was negatively correlated with the viral load showed significant enrichment in viral recognition and cytokine-mediated innate immune GO biological processes (*p*-value range: 4.65 × 10^−10^ to 2.24 × 10^−6^). The 153 genes whose expression was positively correlated with the viral load showed significant enrichment in cholesterol homeostasis, extracellular matrix, and MAPK/ERK pathway-related GO biological processes (*p*-value range: 5.06 × 10^−5^ to 6.53 × 10^−4^). Overall, our results strongly suggest that AT2s’ pre-infection innate immunity and metabolic state affect their susceptibility to SARS-CoV-2 infection and viral load.

## 1. Introduction

Coronavirus disease 2019 (COVID-19), caused by the severe acute respiratory syndrome coronavirus 2 (SARS-CoV-2), manifests through a broad spectrum of symptoms and symptom severity; many who were infected remained asymptomatic (20–40%), and for those who developed symptoms, the disease ranged from mild cold-like symptoms to severe illness and even death [1,2]. This heterogeneity in disease susceptibility and severity of illness (SOI) has been common for all SARS-CoV-2 variants. The major risk factors for severe disease have been age, sex, and the presence of comorbidities such as cardiovascular disease, diabetes, hypertension, and obesity. The geographical location, social vulnerabilities, race, and ethnic differences were also suggested to influence the disease [3]. However, most of the variation in disease susceptibility and SOI remains poorly understood.

SARS-CoV-2 first infects and replicates in the ACE2-expressing cells of the upper respiratory tract and then spreads to the alveolar epithelial cells in the distal lung [4,5]. The viral load in the nasopharynx usually peaks within the first week of infection and was reported to be similar in both asymptomatic and symptomatic patients [6,7]. However, a faster viral clearance was reported in asymptomatic individuals than in those who were symptomatic [8]. Studies of the host response to SARS-CoV-2 infection in the upper respiratory tract using in vitro cell models and patients’ nasopharyngeal swabs have shown that SARS-CoV-2 instigates a strong innate immune response via pathogen recognition receptor (PRR)-activated expression of the interferons (IFNs) and IFN-stimulated genes (ISGs), albeit with delayed kinetics, and PRR/IFN response failed to curtail viral replication in some cases [9,10,11]. Autoantibodies against type I IFNs in the blood of some patients before SARS-CoV-2 infection have been associated with severe COVID-19 [12]. Furthermore, in human in vitro cell models of SARS-CoV-2 infection, pretreatment with exogenous IFNs or a rhinovirus (RV) infection-induced IFN/ISG response blocked SARS-CoV-2 infection. However, exogenous IFNs added post-SARS-CoV-2 infection were significantly less effective in stopping the viral replication, and SARS-CoV-2 proteins inhibited various steps in endogenous IFN production and response [9,11,13,14]. Nevertheless, the spread of SARS-CoV-2 infection in the distal lung and associated damage to the alveolar epithelial cells in severe COVID-19 cases cause acute respiratory distress syndrome (ARDS), the predominant cause of COVID-19-related deaths. An imaging mass cytometry analysis of COVID-19 patients’ postmortem lung tissue showed that SARS-CoV-2 predominantly infects alveolar epithelial cells in the lung, and damage to alveolar epithelial type 2 cells (AT2s) was positively correlated with clinical factors such as organizing pneumonia, the number of days that elapsed since symptom onset, hospitalization and intubation, and lung weight at death [15], suggesting a critical role of these cells in COVID-19 pathology. The AT2s constitute 10–15% of all alveolar cells and are critical for alveolar homeostasis and preventing alveolar collapse in a number of ways, including (1) the production of surfactant protein (S-protein; SP); (2) stabilization of the airway epithelial barrier; (3) immune defense; and (4) regeneration of distal lung epithelial cells through their progenitor function [16,17,18]. Lineage-tracing models have demonstrated that AT2s proliferate and differentiate into alveolar epithelial type 1 cells (AT1s), both in lung injury and in normal unstressed repair [16,19]. Damage to or dysregulated AT2 function has been implicated in pulmonary fibrosis [20]. AT2s express ACE2 and transmembrane serine protease 2 (TMPRSS2), both of which the virus uses for entry into the cell [4,21].

Consistent with the above line of evidence, COVID-19 patients who develop ARDS show a high degree of tissue damage and dysregulated tissue repair with a high level of fibrosis compared to non-COVID-19-associated ARDS and lung injuries [15]. Furthermore, a high level of pulmonary fibrosis is one of the most severe complications that causes permanent lung damage in COVID-19 patients, plausibly directly implicating AT2s’ dysregulated function [22]. Despite significant improvement in our understanding of COVID-19 lung pathology and the cellular landscape involved, in the last few years, factors influencing the disease’s susceptibility and SOI remain largely unidentified.

The heritability estimates of the predicted COVID-19 phenotypic variance in the TwinsUK cohort were reported at 31%, which suggests a significant contribution of host genetic factors to the disease variability [23]. However, like other complex diseases, genome-wide association study (GWAS)-identified COVID-19 risk loci/variants (reviewed in Cappadona et al., 2023; [24]) explain a substantially small portion of the COVID-19 phenotypic variance, with only about 6.5% of the COVID-19 symptom heritability explained by common variants [25]. Therefore, alternative approaches are necessary to identify the genetic determinants of COVID-19 susceptibility and SOI.

In this study, we performed a transcriptome-wide analysis of the cellular response to SARS-CoV-2 infection challenge in AT2s derived from the induced pluripotent stem cells (iPSCs) of 10 individuals of our longitudinal Mexican American Family Study (MAFS) to understand the mechanisms of the AT2′s capacity to regulate SARS-CoV-2 infection and cellular viral load.

## 2. Materials and Methods

Validated iPSC lines reprogrammed from the lymphoblastoid cell lines of 10 MAFS participants using a methodology we have published previously [26,27] were used for the AT2 generation.

### 2.1. AT2 Differentiation

To generate AT2 lines, first, the cryopreserved iPSC lines were expanded in feeder-free conditions and commercially available mTeSR™1 medium (Stem Cell Technologies Inc., Cambridge, MA, USA) and then differentiated into AT2s using a four-stage differentiation protocol adapted from Gotoh et al. (2014) and Jacob et al. (2017 and 2019) [28,29,30]. In the first stage (days 0–4), high-quality iPSCs were differentiated into multipotent definitive endoderm (DE) cells using a commercially available STEMdiff™ Definitive Endoderm Kit (Stem Cell Technologies Inc., Cambridge, MA, USA). In stage 2 (days 5–8), highly enriched multipotent DE cells (>97% cells expressing SOX17 and CXCR4 endodermal markers) were differentiated into anterior foregut endoderm cells (AFECs) in an alveolar epithelial cell (AEC) basal medium containing 3:1 IMDM and Ham’s F12 Nutrient Mix, 1X N2 supplement, 1X B27 supplement, 2 mM GlutaMAX, 0.1% (*w*/*v*) bovine serum albumin Fraction V, 0.05 mg/mL L-ascorbic acid, 50 U/mL penicillin/streptomycin (all from Thermo Fisher Scientific, Waltham, MA, USA), and 0.4 mM monothioglycerol (MilliporeSigma, St. Louis, MO, USA) and supplemented with 100 ng/mL of human recombinant Noggin (Thermo Fisher Scientific, Waltham, MA, USA) and 10 μM TGFβ receptor inhibitor SB-431542 (Bio-Techne Corporation, Minneapolis, MN, USA). In the third stage (days 9–13), highly enriched AFECs (>95% cells expressing SOX2 and FOXA2) were differentiated into ventralized anterior foregut endoderm cells (vAFECs)/ lung progenitor cells in the AEC basal medium (as described above) supplemented with 50 ng/mL of human recombinant BMP4 (Thermo Fisher Scientific, Waltham, MA, USA), 100 nM all-trans retinoic acid, and 3 μM CHIR99021 (MilliporeSigma, St. Louis, MO, USA). On day 14, one part of the generated vAFECs was characterized by immunocytochemistry (ICC) analysis of NKX2-1 and CPM markers to assess differentiation efficiency. Magnetic activated cell sorting (MACS) of CPM-positive cells was performed if necessary to achieve a highly enriched NKX2-1-positive vAFEC population, as described in Gotoh et al. (2014) [28]. In stage 4 (days 14–21), NKX2-1-enriched vAFECs were resuspended in growth factor reduced Matrigel matrix (Corning, Glendale, AZ, USA) at a cell density of ~350 cells/μL, seeded as 3D slabs in 6-well cell culture plates, and then cultured in AT2 medium containing AEC basal medium (described above), 1X HEPES buffer, 0.8 mM CaCl_2_, 3 μM CHIR99021, 50 nM dexamethasone, 0.1 mM 8-Bromoadenosine 3′, 5′-cyclic monophosphate sodium salt, 0.1 mM 3-Isobutyl-1-methylxanthine (all from Millipore Sigma, St. Louis, MO, USA), and 25 ng/mL human recombinant KGF (Thermo Fisher Scientific, Waltham, MA, USA). The alveolosphere outgrowths containing AT2s develop in the 3D Matrigel matrix within 3–7 days. On days 21–25, the differentiated AT2s were harvested by sequential digestion with 1 U/mL dispase (Stem Cell Technologies Inc., Cambridge, MA, USA) and 0.05% trypsin (Thermo Fisher Scientific, Waltham, MA, USA) and were re-seeded as a monolayer (~1.5 × 10^5^ cells/cm^2^) on Matrigel matrix-coated tissue culture plates for ICC characterization and SARS-CoV-2 infection assays.

### 2.2. SARS-CoV-2 Infection Assay

AT2 monolayer cultures were maintained in AT2 medium (described above) until 60 to 70% confluent and then transferred to the University of Texas Rio Grande Valley (UTRGV) Biosafety Level 3 (BSL3) containment laboratory. All SARS-CoV-2 viral procedures were performed in compliance with containment procedures in the BSL3 as approved by UTRGV’s Institutional Biosafety Committee and the Department of Environmental Health, Safety, and Risk Management, under an approved protocol (2019-003-HBA). For the SARS-CoV-2 infection assay, the SARS-CoV-2 USA-WA1/2020 viral strain was obtained from the World Reference Center for Emerging Viruses and Arboviruses at the University of Texas Medical Branch, Galveston, TX (a generous gift from Dr. Kenneth Plante), and a working stock of 6–7 log_10_ of the virus was generated following the method described in Harcourt et al. (2020) [31]. For each sample, infections were performed in triplicate with a multiplicity of infection (MOI) = 10, and the other three replicates were mock-infected (treated with medium without virus). After 2 h of viral inoculation, viral and mock media were replaced with fresh AT2 medium, and cells were returned to the cell culture incubator for 48 h. After 48 h, one replicate of each of the mock and SARS-CoV-2 infection-challenged AT2s was fixed using 4% paraformaldehyde solution (Millipore Sigma, St. Louis, MO, USA), and the other two replicates of each of the mock and SARS-CoV-2 infection-challenged cells were harvested by digestion with RNA extraction buffer. The tubes containing cell digest for RNA extraction and the plates of fixed AT2s were sealed, surface decontaminated, and transferred to the BSL2 laboratory for further analysis.

### 2.3. Immunocytochemistry Analysis

All 10 generated AT2 lines were characterized by ICC analysis of the AT2 markers NKX2-1, EpCAM, SFTPB, and SFTPC before the SARS-CoV-2 infection assays. AT2s in both 3D alveolosphere culture during culture expansion and in monolayer culture prior to the viral infection challenge were analyzed using the standard ICC techniques, and the primary antibodies for NKX2-1 (rabbit anti-human NKX2-1, MAB94581, R&D Systems, Bio-Techne Corporation, Minneapolis, MN, USA), EpCAM (mouse anti-human EpCAM, MA5-13917, Invitrogen, ThermoFisher Scientific, Waltham, MA, USA), SFTPB (mouse anti-human SFTPB, MA1-204, Invitrogen, ThermoFisher Scientific, Waltham, MA, USA), and SFTPC (rabbit anti-human SFTPC, PA5-71680, Invitrogen, ThermoFisher Scientific, Waltham, MA, USA) and secondary antibodies donkey anti-mouse Alexa Fluor™ 488 and donkey anti-rabbit Alexa Fluor™ 594 (R37114 and R37119, respectively; Invitrogen, ThermoFisher Scientific, Waltham, MA, USA). ICC analysis of the SARS-CoV-2-infected cells in replicate 1 (R1) of the viral infection assay was performed using a primary antibody for the SARS-CoV-2 nucleocapsid protein (rabbit anti-SARS-CoV-2 Nucleocapsid Protein (HL344), mAb #26369, Cell Signaling Technology, Danvers, MA, USA) and a secondary antibody donkey anti-rabbit Alexa Fluor™ 594 (R37119; Invitrogen, ThermoFisher Scientific, Waltham, MA, USA). In each ICC analysis, cell nuclei were counterstained with DAPI (4′, 6-diamidino-2-phenylindole; Invitrogen), and appropriate negative controls were included. Cells were imaged immediately after ICC staining using a Carl Zeiss Axio Imager D2 research microscope (Carl Zeiss Microscopy, LLC, White Plains, NY, USA) and/or a PerkinElmer Operetta high-content screening system (PerkinElmer, Waltham, MA, USA). Quantitative measurements of the cells expressing AT2 markers (cells positive for AT2 markers) prior to the viral infection assay and the number of cells infected with SARS-CoV-2 (SARS-CoV-2 nucleocapsid protein-positive cells) postviral infection were performed using the PerkinElmer Operetta high-content screening (HCS) system and Harmony software v4.1 (PerkinElmer, Waltham, MA, USA).

### 2.4. RNA Extraction, RT-PCR, and RNA Sequencing

Total RNA was extracted from each of the mock and SARS-CoV-2 infection-challenged AT2s (2 replicates each of the mock- and SARS-CoV-2-infected cells per sample) using a commercially available RNeasy Mini Kit (Qiagen, Germantown, MD, USA) and the manufacturer’s protocol. RNA quality and quantity were assessed using a NanoDrop 2000 Spectrophotometer (Thermo Fisher Scientific, Waltham, MA, USA) and an Agilent 2200 TapeStation system (Agilent, Santa Clara, CA, USA).

Relative quantification of the viral load in the SARS-CoV-2-infected replicates 2 and 3 (R2 and R3) was performed by RT-PCR analysis using primers and probe sequences specific to the SARS-CoV-2 nucleocapsid protein gene, as described in the US CDC rRT-PCR panel N1 assay in Lu et al. (2020) [32]. The primers and probe were synthesized and obtained commercially (Eurofins Genomics Company, Louisville, KY, USA). The measured viral RNA quantities were normalized by the expression of the human housekeeping gene GAPDH as an endogenous control (Applied Biosystems, ThermoFisher Scientific, Waltham, MA, USA). The RT-PCR was performed using TaqPath™ 1-Step multiplex master mix and on a QuantStudio™ 3 Real-Time PCR instrument (ThermoFisher Scientific, Waltham, MA, USA).

RNA sequencing of both mock and SARS-CoV-2 infection-challenged replicates (2 replicates each per sample) was performed using the Illumina Stranded mRNA Prep Ligation Kit on an Illumina NovaSeq6000 instrument. Briefly, 1µg of high-quality total RNA per sample per replicate and the reagents supplied in Illumina Stranded mRNA sample preparation kit v2 (Illumina, Inc., San Diego, CA, USA) were used to prepare mRNA sequencing libraries. Oligo-dT magnetic beads supplied in the kit were used to first enrich the poly-A tail containing mRNA molecules from the total RNA. Divalent cations and elevated temperature were then used to fragment the enriched mRNA into ~200–600 base pair-sized molecules. The first-strand cDNA was synthesized from cleaved RNA fragments using reverse transcriptase and random primers, followed by second-strand cDNA synthesis using DNA polymerase-I and RNase H. The synthesized cDNA fragments were end-repaired, and adaptors were ligated. The resulting cDNA libraries were purified, enriched by PCR, and then deep sequenced on an Illumina NovaSeq6000 instrument.

### 2.5. RNA Sequencing Analyses

The Illumina bcl2fastq2 software v2.20.0 (Illumina, Inc., San Diego, CA, USA) was used to generate and demultiplex raw fastq sequence files. After pre-alignment QCs, sequences were aligned to human genome assembly GRCh38 (hg38) and mapped to RefSeq transcripts using StrandNGS software v4.1 (Strand Life Sciences Pvt. Ltd., Bangalore, India). The aligned reads were filtered based on default read quality metrics, and log transformation and “DESeq” normalization were applied. Known genes/mRNAs with a normalized read count (NRC) ≥ 10 and conditions as described in the results were considered for various differential gene expression analyses.

### 2.6. Differential Gene Expression Analyses

To identify genes that were significantly differentially expressed (DE) between various conditions described in the results, moderated *t* statistics or one-way ANOVA and expression fold change (FC) analyses were performed. The genes with moderated *t* statistics/one-way ANOVA FDR-corrected *p*-value ≤ 0.05 and FC absolute (FC abs) ≥ 2.0 between the pair(s) of conditions were considered significantly differentially expressed.

### 2.7. Functional Annotations and Enrichment Analyses

Disease/functional annotations and enrichment analyses of the gene sets of interest, identified by the differential gene expression analyses detailed in the results, were performed using the Ingenuity Pathway Analysis (IPA) platform (QIAGEN Digital Insights, Redwood City, CA, USA) and ‘Enrichr’ and ‘ShinyGO v0.77’ gene set enrichment analysis web tools [33,34] (accessed November 2023). In IPA, right-tailed Fisher’s exact test FDR-corrected *p*-values were used for enrichment significance, and the direction of functional change was assessed by the activation *z*-score detailed in Kramer et al. (2014) [35]. Briefly, in IPA activation *z*-score is used to infer activation states of an enriched biological function. The basis of this inference is the relationship between genes and biological function(s) that are literature-derived. The direction of effect (up- or downregulated) is determined by DE genes in the data set and the direction of the gene’s effect on the biological function. A positive *z*-score indicates upregulation, and a negative *z*-score indicates downregulation of the biological function. The ‘Enrichr’ implements several enrichment scores as described in Chen et al. (2013) [33]; we ranked our ‘Enrichr’ results based on computed Fisher exact test *p*-values. In ‘ShinyGO’ enrichment analyses, FDR is calculated based on nominal *p*-value from the hypergeometric test and FDR-corrected *p*-values ≤ 0.05 were used for statistical significance.

## 3. Results

### 3.1. AT2 Generation

**Figure 1:**
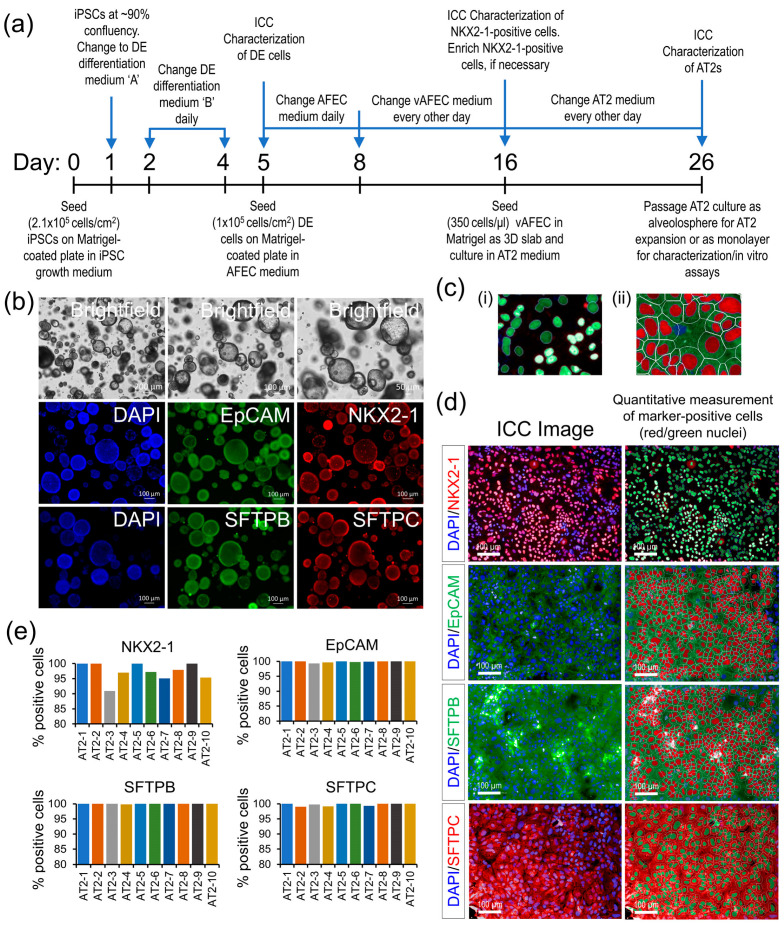
AT2 generation and characterization. (**a**) A schematic outline of the AT2 differentiation protocol. (**b**) Morphological and ICC analysis of the generated AT2s’ 3D alveolospheres showing expression of AT2-specific markers NKX2-1, EpCAM, SFTPB, and SFTPC. (**c**) (i,ii) Representative enlarged images detailing HCS quantification methodology of the nuclear and cytoplasmic expression of AT2 markers: (i) quantification of NKX2-1 nuclear expression, (ii) expression of EpCAM, SFTPB, and SFTPC markers measured in the cytoplasmic area marked by the white line minus the nuclear area. (**d**) Representative ICC images of each AT2 marker expression in monolayer culture; expression of each marker was quantitatively measured, as detailed in ‘c’, and cells passing a fluorescence threshold, set uniformly across all samples, were counted positive for the respective marker and identified as red/green highlighted nuclei. (**e**) Bar graphs showing the percentage of cells positive for each AT2 marker across 10 generated AT2 lines.

Existing iPSC lines from 10 individuals of our longitudinal MAFS were differentiated into AT2s using a four-stage differentiation protocol summarized in Figure 1a, which uses a sequential modulation of the signaling pathways by small molecules and biologicals detailed in the methods. All generated AT2 lines showed high expression of AT2-specific markers NKX2-1, EpCAM, SFTPB, and SFTPC measured by ICC both in 3D alveolosphere and monolayer AT2 cultures (Figure 1b–d). An HCS analysis of the ICC-stained AT2 markers, which quantified five to eight thousand cells across nine randomly chosen visual fields per AT2 line showed that >90% of the generated cells across all 10 AT2 lines expressed NKX2-1, a key lung epithelial cell transcription factor; other AT2-specific markers EpCAM, SFTPB, and SFTPC were expressed in ~99 to 100% of generated cells (Figure 1c,d). These results suggest a highly uniform AT2 differentiation across the 10 samples.

**Figure 2:**
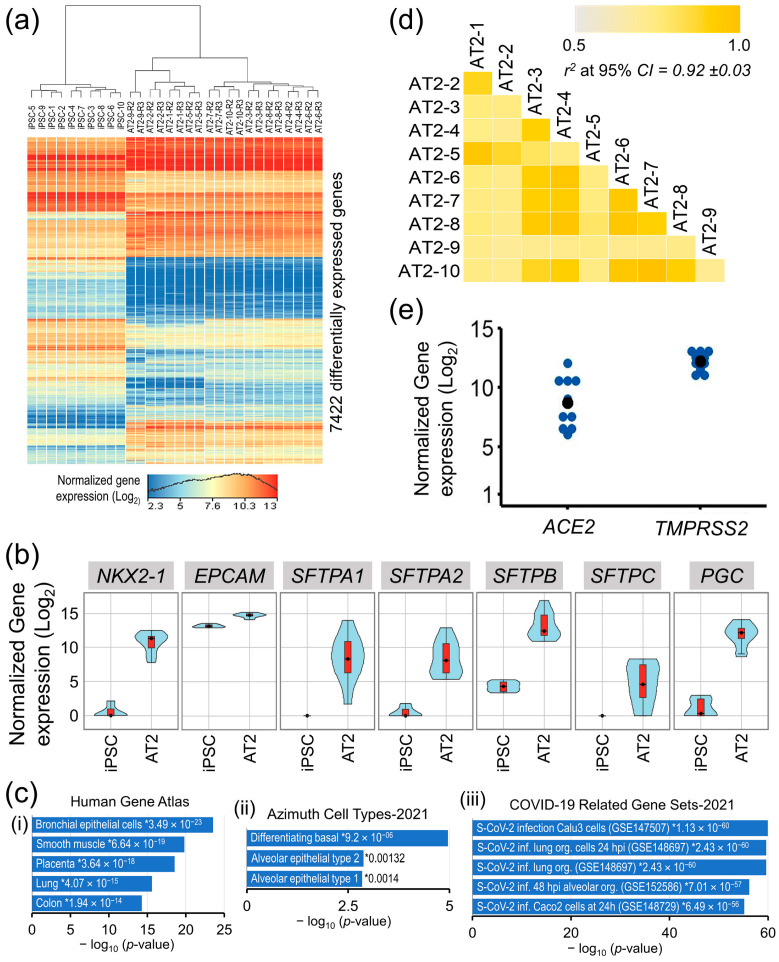
Transcriptomic characterization of the iPSC generated AT2 lines. (**a**) Heat map of genes that were significantly differentially expressed between iPSCs and differentiated AT2 lines. (**b**) Gene expression violin plots of AT2-specific genes in iPSCs and differentiated 10 AT2 lines showing significant upregulation of AT2-specific genes in all generated AT2 lines. (**c**) Gene set enrichment analysis plots of AT2s’ upregulated transcriptome in Human Gene Atlas, Azimuth Cell Types, and COVID-19-related gene sets; * enrichment significance *p*-values. (**d**) Correlation coefficient (*r*^2^) plot based on the 13,285 genes found expressed (NRC ≥ 10) in the generated AT2s. (**e**) Gene expression plot of *ACE2* and *TMPRSS2* in the generated AT2s.

In a differential gene expression analysis between iPSCs’ and AT2s’ (control replicates R2 and R3) expressed transcriptomes (16,038 genes that were expressed (NRC ≥ 10) in iPSCs and/or differentiated AT2 lines), a total of 7422 genes were significantly differentially expressed (moderated *t* statistics FDR-corrected *p*-value ≤ 0.05 and FC *abs* ≥ 2.0) and accounted for ~40% of the iPSC’s and AT2′s expressed transcriptome (Figure 2a).

The expression of well-known AT2-associated genes *NKX2-1*, *EpCAM*, *SFTPA1*, *AFTPA2*, *SFTPB*, *SFTPC*, and *PGC* was significantly upregulated in the generated AT2s (Figure 2b), whereas the expression of pluripotency-associated genes *POU5F1*, *NANOG*, *SOX2*, and *ESRG* was significantly downregulated. A high correlation between all 10 generated AT2 lines expressed transcriptome (13,285 genes having NRC ≥ 10 in all generated AT2 lines), where *r*^2^ [95% CI] = 0.92 ± 0.03, validates a uniform differentiation of the generated AT2s across all samples (Figure 2d). Gene set enrichment analysis of the 3023 genes that were significantly upregulated in the generated AT2s showed significant enrichment in the Human Gene Atlas of the bronchial epithelial cells (*p*-value = 3.49 × 10^−23^) and the lung cells (*p*-value = 4.07 × 10^−15^) and, more specifically, in the gene set of alveolar epithelial type 2 (*p*-value = 0.00132) in the Azimuth Cell Type data base (CL0002063). Additionally, the AT2′s upregulated transcriptome also showed very high enrichment in COVID-19-related gene sets of Calu3 cells (GSE147507) [36], human lung organoids (GSE148697) [37], and human alveolar cell organoids (GSE152586) [38], with *p*-values ranging from 1.13 × 10^−60^ to 7.01 × 10^−57^ (Figure 2c). Furthermore, all generated AT2 lines robustly expressed the *ACE2* and *TMPRSS2* genes that facilitate SARS-CoV-2 entry into the lung alveolar epithelial cells (Figure 2e).

### 3.2. Individual-Specific Variation in AT2s’ Susceptibility to SARS-CoV-2 Infection and Viral Load

**Figure 3:**
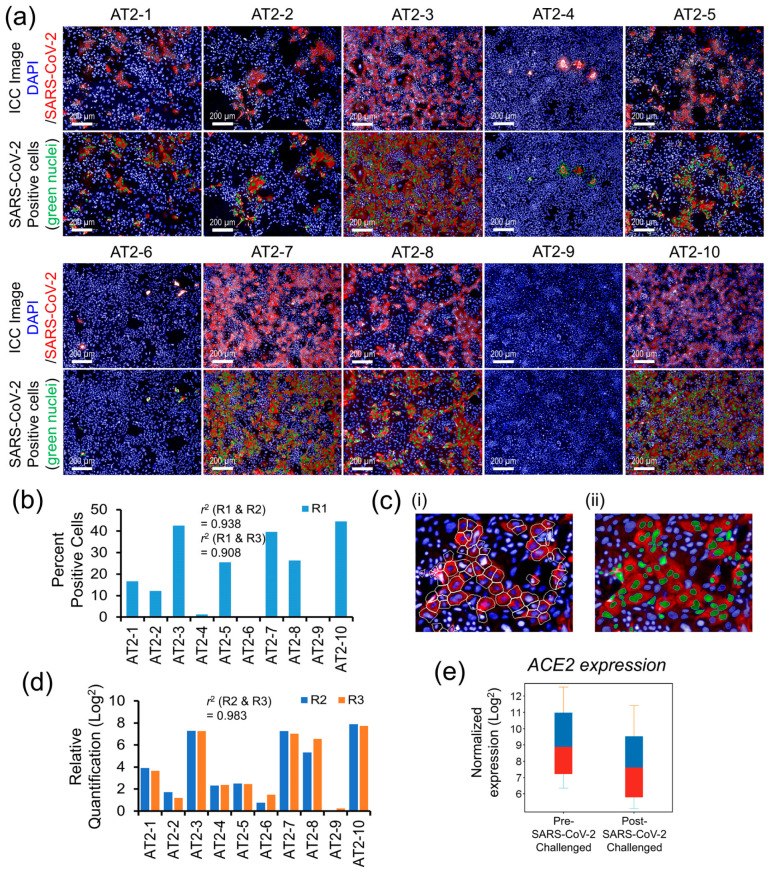
Interindividual variability in AT2s’ susceptibility to SARS-CoV-2 infection and viral load. (**a**) Post SARS-CoV-2 infection challenge ICC-based HCS quantification of SARS-CoV-2 nucleocapsid protein-positive AT2s (a total of 9 randomly chosen visual fields per AT2 line were analyzed for the quantitative measures of SARS-CoV-2-positive cells). (**b**) Comparative bar graph of percent of SARS-CoV-2-positive cells across 10 AT2 samples quantified by HCS analysis of the SARS-CoV-2-infected replicate R1. (**c**) Representative enlarged images detailing HCS quantification methodology; (i) SARS-CoV-2 nucleocapsid protein-associated ICC fluorescence was measured in the cell cytoplasm of each cell marked by the white cell boundary minus nuclear area; (ii) cells passing a fluorescence threshold, set uniformly across all samples, were counted positive for SARS-CoV-2 infection and are represented by green nuclei in the image. (**d**) Comparative bar graph of RT-PCR-based relative quantification of the SARS-CoV-2 nucleocapsid protein gene expression (viral load) across 10 AT2 samples (measurements were performed on SARS-CoV-2-infected replicates R2 and R3 of all 10 AT2 lines). (**e**) Change in *ACE2* gene expression between mock and SARS-CoV-2 infection-challenged AT2s.

Quantitative measures of the AT2s’ susceptibility to SARS-CoV-2 infection and the postinfection viral load are shown in Figure 3a–d. The replicates R1 of each AT2 line, both after the mock and SARS-CoV-2 infection challenge, were analyzed by ICC labeling of the SARS-CoV-2 nucleocapsid protein (NP) and HCS quantification of the NP-positive (SARS-CoV-2-infected) cells as a surrogate measure of AT2s’ susceptibility to SARS-CoV-2 infection (Figure 3a,b). The NP’s ICC fluorescence was quantified in the cell cytoplasm, and the cells that passed a set NP ICC fluorescence threshold across all AT2 lines were counted as SARS-CoV-2-infected, as detailed in Figure 3c(i,ii). The other two replicates (R2 and R3) were quantified for NP gene expression as a measure of cellular viral load after the mock and SARS-CoV-2 infection challenge using RT-PCR. Both HCS-based quantitative measures of SARS-CoV-2-infected cells and the RT-PCR-based measures of the viral load showed high variation across the AT2 lines of 10 individuals, suggesting a significantly high individual-specific differential response to SARS-CoV-2 infection challenge (Figure 3b,d). Additionally, quantitative measures of infection susceptibility and viral load were highly reproducible (similar) between replicates (*r*^2^ = 0.938 between R1 and R2; 0.908 between R1 and R3; and 0.983 between R2 and R3), suggesting that the donor-specific variation observed in SARS-CoV-2 infection-induced cellular response was likely due to biological factors. The expression of *ACE2* was significantly downregulated (*FC* = 2.64) postinfection (Figure 3e) and showed a high correlation (*r^2^* ~0.70) with the viral load measures.

### 3.3. AT2s’ Transcriptomic Response Was Highly Correlated with SARS-CoV-2 Infection and Viral Load Measures

**Figure 4:**
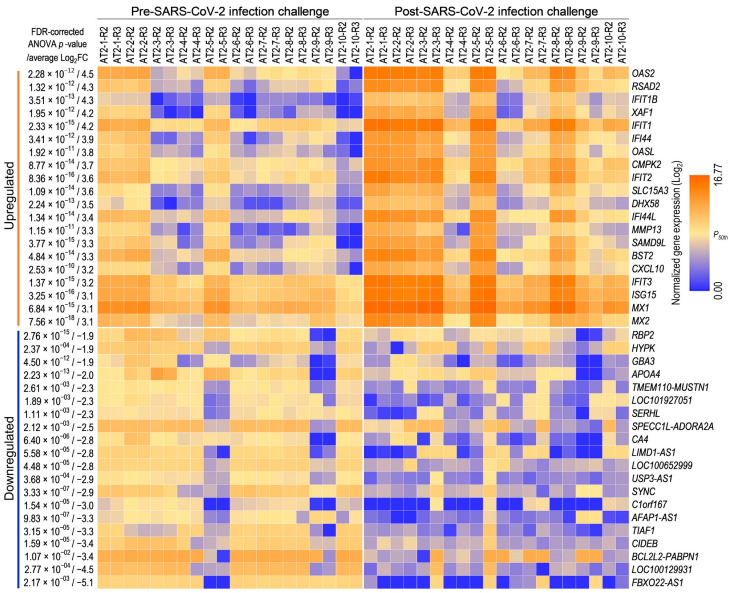
AT2s’ transcriptomic response to SARS-CoV-2 infection: expression heat map of the top and bottom 20 genes that were significantly up- or downregulated between mock and SARS-CoV-2 infection-challenged AT2s.

To investigate the transcriptome-wide AT2 cellular response to SARS-CoV-2 infection, 15,640 genes with an NRC ≥ 10 in at least 50% of the replicates were considered expressed. A pairwise differential gene expression analysis between mock and SARS-CoV-2 infection-challenged replicates across 10 AT2 lines (two replicates per condition per line) using these expressed genes identified 1393 genes that were significantly (one-way ANOVA FDR-corrected *p* ≤ 0.05; FC *abs* ≥ 2.0) DE between mock and SARS-CoV-2 infection-challenged AT2s (Table S1). The SARS-CoV-2 infection-challenged expression change in 712 and 645 DE genes showed >25% correlation (*r*^2^ absolute > 0.25) with the cellular measures of SARS-CoV-2-infection and viral load, respectively. The top and bottom 20 genes whose expression was either significantly upregulated or downregulated (highest average FC *abs* across 10 samples) suggest upregulation of the innate immune response and downregulation of metabolic processes in SARS-CoV-2 infection-challenged AT2s (Figure 4).

**Figure 5:**
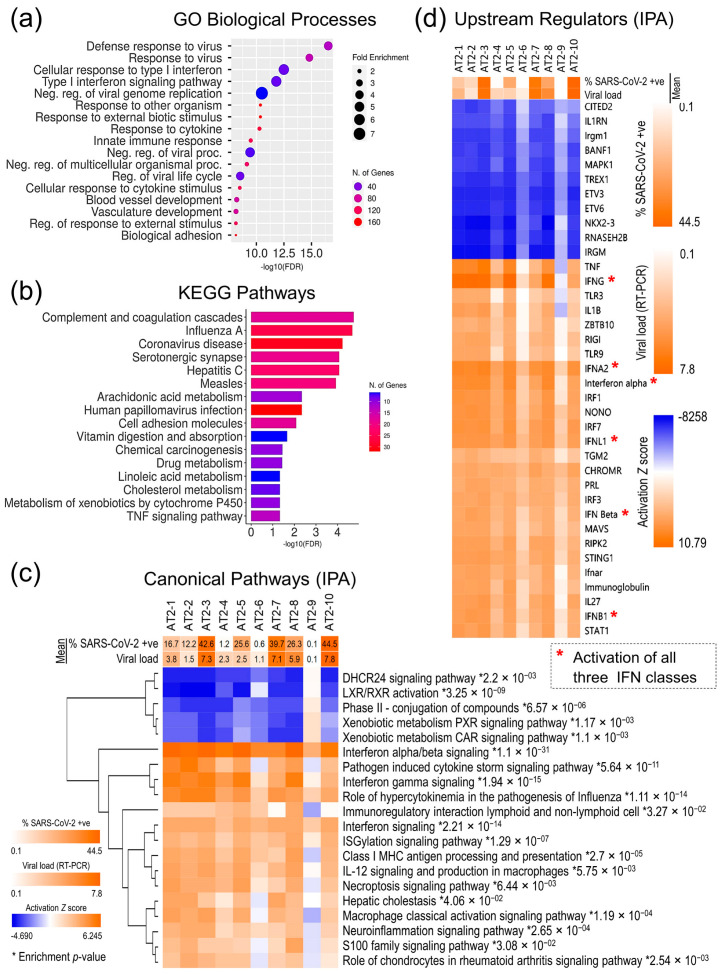
AT2s’ cellular response to SARS-CoV-2 infection. (**a**) Top 20 GO biological processes that were significantly enriched in 1393 DE genes. (**b**) Top 20 KEGG pathways that were significantly enriched in 1393 DE genes. (**c**) Activation *z*-score heatmap of top 20 IPA canonical pathways, which were significantly enriched and differentially regulated post-SARS-CoV-2 infection in 10 AT2 lines; * enrichment significance *p*-values. (**d**) Activation *z*-score heatmap of the upstream regulators that were predicted to be significantly up- or downregulated based on the 1393 DE genes.

For a more comprehensive characterization of the AT2 transcriptomic response, functional annotation and enrichment analyses of all the 1393 DE genes in gene ontology (GO) biological processes, Kyoto Encyclopedia of Genes and Genomes (KEGG) pathways, and IPA canonical pathways were performed, which further validates that SARS-CoV-2 infection induced a strong innate immune response in AT2s and viral infection-related cellular innate immune response pathways were significantly enriched (*p*-value ≤ 0.05) and highly upregulated (*z*-score ≥ 3.5), whilst the cholesterol- and xenobiotic-related metabolism pathways were significantly downregulated (Figure 5a–c). Similarly, the repertoire of upstream regulators that were significantly enriched and predicted to be differentially regulated in SARS-CoV-2 infection-challenged AT2s (*p*-value ≤ 0.01; activation *z*-score-absolute ≥ 6) also showed significant upregulation of the interferon regulatory factors (IRFs: *IRF1*, *IRF3*, and *IRF7*) and their downstream interferons (IFNs) and IFN-stimulated genes (ISGs). These results strongly suggest that AT2s mount a strong primary antiviral innate immune response against SARS-CoV-2 infection, which plausibly plays a role in clearing the viral infection. The AT2′s innate immune response to SARS-CoV-2 infection across 10 lines was directly proportional to the measures of SARS-CoV-2 infection susceptibility and postinfection cellular viral load. However, it is not clear if post-SARS-CoV-2 infection response played a causal role in the interindividual variability observed in the infection susceptibility and/or viral load (Figure 5c,d).

### 3.4. AT2s’ Pre-Infection Innate Immunity Attenuates Susceptibility to SARS-CoV-2 Infection and Viral Load

Interestingly, the pre-infection (mock-challenged) expression of 238 genes that were DE between mock and SARS-CoV-2 infection-challenged AT2s showed a statistically significant correlation with the SARS-CoV-2 viral load measures (FDR-corrected *p*-value ≤ 0.05 and *r*^2^-absolute ≥ 0.57) across 10 AT2 lines (Table S2 and Figure S1). The pre-infection expression of these genes also showed a high correlation (*r*^2^ absolute ≥ 0.30) with the measure of SARS-CoV-2 infection susceptibility (SARS-CoV-2 NP-positive cells) in the 10 AT2 lines. The 85 genes whose expression was significantly negatively correlated with the viral load and infection susceptibility showed significant enrichment in viral recognition and cytokine-mediated innate immune response GO biological processes (*p*-value range: 4.65 × 10^−10^ to 2.24 × 10^−6^). The GO biological processes associated with negative regulation of the viral processes were also significantly (*p*-value ≤ 6.07 × 10^−6^) enriched (Table 1). The 153 genes whose expression was significantly positively correlated with the viral load showed significant enrichment in lipid homeostasis-related (*p*-value range: 5.06 × 10^−5^ to 5.73 × 10^−4^), extracellular matrix (ECM)-related (*p*-value range: 6.42 × 10^−5^ to 4.19 × 10^−4^), and MAPK/ERK pathway-related (*p*-value range: 4.63 × 10^−4^ to 6.53 × 10^−4^) GO biological processes (Table 1).

The expression of the transmembrane serine protease 6 (*TMPRSS6*) gene in the mock infection-challenged AT2s showed the highest correlation (*r*^2^ = 0.918; FDR-corrected *p*-value = 1.51 × 10^−5^) with SARS-CoV-2 viral load measures. Serine proteases, *TPMRSS2* in particular, are already known to facilitate SARS-CoV-2 spike protein fusogenic activity and virus entry into host cells.

## 4. Discussion

COVID-19 phenotypic variance has a significant genetic basis; however, factors that explain disease risk beyond age, sex, and cardiovascular and metabolic comorbidities are yet to be fully identified [24]. In part, this is due to the complex etiology of the COVID-19 disease and difficulty in sampling certain disease states, less severe and nonsymptomatic cases in particular. Nonetheless, ARDS remained the predominant cause of COVID-19-related deaths, and AT2s have emerged as one of the primary cell types affected [15,39,40,41]. In this study, we investigated AT2s’ capacity to regulate SARS-CoV-2 infection and cellular viral load as surrogate measures of COVID-19 phenotypic variability using iPSC-derived AT2s from 10 different individuals and a transcriptome-wide approach. Our comprehensive transcriptomic and functional annotation analysis of the generated AT2s (Figure 1 and Figure 2) and previously published studies validate that the human iPSC-derived AT2s possess a transcriptomic and functional profile that is very similar to human primary AT2s and therefore are the close surrogates and the relevant cells to model SARS-CoV-2 infection-induced lung damage [29,42,43]. To the best of our knowledge, our SARS-CoV-2 infection challenge of the generated 10 AT2 lines in in vitro cultures for the first time shows individual-specific variability in the AT2s’ susceptibility to SARS-CoV-2 infection and postinfection cellular viral load, which is highly reproducible across replicates (Figure 3). This observed variability in the iPSC-derived AT2s’ susceptibility to SARS-CoV-2 infection and cellular viral load plausibly recapitulates the COVID-19 phenotypic variability in disease susceptibility and SOI. Given that iPSC-derived cells are minimally influenced by life-experienced environmental exposures, it is highly likely that the observed interindividual variation in AT2 response to SARS-CoV-2 infection is driven by genetic factors [44,45,46].

SARS-CoV-2 infection induced a strong primary immune response in AT2s, RNA virus sensing PRRs *DDX58* (also known as RNA sensor RIG-I or RIGI) and *IFIH1* (also known as melanoma differentiation-associated gene 5 or MDA-5), PRR-induced IFN regulatory factors (*IRF1*, *IRF3*, and *IRF7*), and their downstream IFNs (type I: *INF-α/β*; type II: *IFN-γ*; and type III: *IFN-λ*), and ISGs were upregulated or predicted to be upregulated (activation *z*-score ≥ 2.0) in SARS-CoV-2 infection-challenged AT2s (Table S1 and Figure 5c,d). Contrary to the findings in a previous report [47], our data strongly suggest induction of all three IFN classes (type I, II, and III) in the AT2s’ SARS-CoV-2 infection response, albeit with high interindividual heterogeneity, the IFNs/ISGs activation was directly proportional to the measures of cellular viral load and percentage of infected cells (Figure 5c,d). However, transcriptomic expression of the IFN genes (*IFNB1*, *IFNL1*, *IFNL2*, and *IFNL3*) was detected only in SARS-CoV-2-infected AT2 lines, which had a higher viral load and did not pass the threshold set for the expressed gene in our differential gene expression analysis. Interindividual heterogeneity in SARS-CoV-2 infection response in peripheral blood mononuclear cells and viral load-associated tropism to IFN response in postmortem lung tissue has also been reported in recent studies [48,49].

Whilst the effect of post-SARS-CoV-2 infection innate immune response on the infection susceptibility and postinfection viral load in AT2s is not clear, AT2s’ pre-infection innate immunity showed a significant inverse relationship with SARS-CoV-2 infection susceptibility and postinfection viral load. The pre-infection expression of 85 genes that were also DE post-SARS-CoV-2 infection was significantly negatively correlated with postinfection viral load and infection susceptibility in the 10 AT2 lines. The significant enrichment of these genes in viral recognition and cytokine-mediated innate immune GO terms (Table 1) strongly suggest that pre-infection interindividual variability in the innate immune processes is causally associated with SARS-CoV-2 infection susceptibility and postinfection viral load and plausibly plays a role in COVID-19 susceptibility and SOI. This antagonism between pre-infection innate immunity and COVID-19 susceptibility and SOI has also been suggested in children’s upper airway epithelial cells, macrophages, and dendritic cells [10]. Furthermore, heterologous IFNs/ISGs response to prior RV infections has been reported to suppress SARS-CoV-2 replication throughout the epithelium in in vitro experiments [9]. Pre-activated innate immunity likely preempts IFN/ISG induction before SARS-CoV-2-expressed proteins antagonize and delay such response.

Interestingly, the pre-infection expression of 153 genes, which were also DE post-SARS-CoV-2 infection, correlated significantly positively with post-SARS-CoV-2 infection viral load and infection susceptibility (Table S2). The significant enrichment of these genes in cholesterol homeostasis, ECM, and MAPK/ERK pathway-related GO terms (Table 1) suggest that these metabolic processes may have an agonistic role in SARS-CoV-2 viral infection and replication, while a plethora of evidence, including our own findings in AT2s, shows that, postinfection, SARS-CoV-2 inhibits host cholesterol metabolism (reviewed in [50,51]). Increasing evidence also supports the view that the cholesterol- and sphingolipids-rich lipid rafts play a vital role in SARS-CoV-2 infection. Intriguingly, the SARS-CoV-2 spike (S) protein contains putative cholesterol recognition amino acid consensus motifs, which can directly interact with cholesterol and an antibody, blocking the putative cholesterol binding site of the SARS-CoV-2 S protein and strongly inhibiting SARS-2-S pseudovirus infection [52]. Furthermore, two independent CRISPR screens showed that cholesterol metabolism genes including the genes involved in sterol metabolism are essential for SARS-CoV-2 infection [53,54]. Also, the human lung Calu-3 cells treated with high cholesterol developed a higher SARS-CoV-2 viral load [55]. A pronounced remodeling of ECM was reported in COVID-19 cases [56,57]. However, the role of ECM in SARS-CoV-2 infection susceptibility and viral replication is enigmatic. The ADAMTS proteins may play a role in post-SARS-CoV-2 infection fibrosis and regulation of the TGF-β pathway [58]. However, their role in infection susceptibility and/or postinfection viral load is not clear. A higher protein level of MMP10 in the serum of nonsymptomatic SARS-CoV-2-infected individuals was reported in one study [59]. However, *MMP10* gene expression was positively correlated with SARS-CoV-2 viral load and showed a significant downregulation in post-SARS-CoV-2 infection in our study. As stated in the results, the pre-infection expression of the *TMPRSS6* gene showed the highest positive correlation with SARS-CoV-2 viral load in 10 AT2 lines. The *TMPRSS6* is a liver serine protease that negatively regulates hepcidin [60,61,62,63]. A higher level of hepcidin suggests a pro-inflammatory state [64,65], which is likely less conducive to SARS-CoV-2 infection and/or viral replication. Alternatively, a lower hepcidin level may promote viral infection and replication. What significance these connections might hold needs further study. Pre-infection positive regulation of MAPK-ERK1/2 pathways showed a positive correlation with AT2s’ susceptibility to SARS-CoV-2 infection and postinfection viral load. A recent study showed that treatment of Caco-2 cells with SARS-CoV-2 spike protein’s receptor binding domain activated the key mediators (ERK1/2 and CRAF) of MAPK signaling, and inhibition of MAPK signaling by MEK inhibitors reduced SARS-CoV-2 infection [66].

## 5. Conclusions

Overall, our study demonstrates that human iPSC-derived AT2s are transcriptionally and functionally very similar to human primary AT2s and therefore are close surrogates to investigate interindividual variability in SARS-CoV-2-induced lung damage. Our results for the first time show donor-specific variability in iPSC-derived AT2s’ susceptibility to SARS-CoV-2 infection and postinfection viral load. This observed variability in the iPSC-derived AT2s’ susceptibility to SARS-CoV-2 infection and viral load plausibly recapitulates the COVID-19 phenotypic variability observed in patients. We report a comprehensive list of 1393 genes that constituted AT2s’ response to SARS-CoV-2 infection and show significant induction of AT2s’ innate immunity pathways and downregulation of cholesterol and xenobiotic metabolic pathways. More interestingly, our results show that AT2s’ pre-infection innate immunity and metabolic state significantly affected their susceptibility to SARS-CoV-2 infection and viral load. There are a few important caveats to our study. We focused only on AT2s’ cellular response to SARS-CoV-2 infection, a cell type primarily affected in SARS-CoV-2 distal lung infection. The SARS-CoV-2 infection challenge was performed in AT2 in vitro cultures. However, other cell types including the likely recruitment of immune cells in the infection response may confound the AT2 response in COVID-19 patients. The iPSC-derived cells are minimally influenced by life-experienced environmental exposures; it is highly likely that the observed interindividual variation in AT2 response to SARS-CoV-2 infection is driven by genetic/biological factors but lacks environmental influence. Lastly, we did not have COVID-19 data on individuals whose samples were used for iPSC derivation.

## Figures and Tables

**Table 1:** GO biological processes (terms) that were significantly enriched in the 238 genes whose expression was highly correlated with post-SARS-CoV-2 infection viral load in AT2 lines.

GO Biological Processes	*p*-Value	Odds Ratio	Genes
i. Top 10 GO terms enriched in 85 genes whose pre-infection expression was negatively correlated with viral load			
Defense response to virus (GO:0051607)	4.65 × 10^−10^	16.48	*IFIH1*; *DDX58*; *CD40*; *OAS1*; *OAS3*; *STAT2*; *DHX58*; *IRF7*; *DDX60L*; *TNF*; *TLR3*; and *PML*
Cytosolic pattern recognition receptor signaling pathway (GO:0002753)	3.90 × 10^−8^	69.09	*IFIH1*; *DDX58*; *OAS3*; *DHX58*; *IRF7*; and *TNFAIP3*
Defense response to symbiont (GO:0140546)	2.21 × 10^−7^	14.68	*IFIH1*; *DDX58*; *CD40*; *OAS1*; *OAS3*; *STAT2*; *IRF7*; *DDX60L*; and *TLR3*
Positive regulation of type I interferon production (GO:0032481)	3.03 × 10^−7^	26.00	*IFIH1*; *DDX58*; *OAS1*; *OAS3*; *DHX58*; *IRF7*; and *TLR3*
Positive regulation of interferon-beta production (GO:0032728)	4.18 × 10^−7^	40.09	*IFIH1*; *DDX58*; *OAS1*; *OAS3*; *IRF7*; and *TLR3*
Positive regulation of cytokine production (GO:0001819)	1.03 × 10^−6^	8.43	*IFIH1*; *DDX58*; *CD274*; *CD40*; *BTN3A1*; *DHX58*; *IRF7*; *HLA-A*; *PTGS2*; *TNF*; and *TLR3*
Antigen processing and presentation of endogenous peptide antigen (GO:0002483)	1.72 × 10^−6^	57.80	*TAP1*; *HLA-A*; *HLA-F*; and *MICB*
Regulation of interferon-beta production (GO:0032648)	2.24 × 10^−6^	27.60	*IFIH1*; *DDX58*; *OAS1*; *OAS3*; *IRF7*; and *TLR3*
Negative regulation of viral genome replication (GO:0045071)	3.01 × 10^−6^	25.87	*IFIH1*; *OAS1*; *OAS3*; *TNF*; and *N4BP1*
Negative regulation of viral process (GO:0048525)	6.07 × 10^−6^	22.16	*IFIH1*; *OAS1*; *OAS3*; *TNF*; and *N4BP1*
ii. Top 10 GO terms enriched in 153 genes whose pre-infection expression was positively correlated with viral load			
Very-low-density lipoprotein particle assembly (GO:0034379)	5.06 × 10^−5^	56.69	*SOAT2*; *MTTP*; and *APOB*
Extracellular matrix organization (GO:0030198)	6.42 × 10^−5^	6.46	*ADAMTS15*; *POSTN*; *ADAMTS2*; *COL5A1*; *TMPRSS6*; *PLG*; *HMCN1*; and *MMP10*
Cholesterol homeostasis (GO:0042632)	2.27 × 10^−4^	9.97	*SOAT2*; *MTTP*; *NR1D1*; *CYP7B1*; and *APOB*
Sterol homeostasis (GO:0055092)	2.42 × 10^−4^	9.83	*SOAT2*; *MTTP*; *NR1D1*; *CYP7B1*; and *APOB*
Extracellular matrix disassembly (GO:0022617)	3.52 × 10^−4^	13.29	*ADAMTS15*; *TMPRSS6*; *PLG*; and *MMP10*
Water transport (GO:0006833)	3.89 × 10^−4^	24.79	*PDPN*; *SLC5A1*; and *UPK3A*
Cellular component disassembly (GO:0022411)	4.19 × 10^−4^	12.66	*ADAMTS15*; *TMPRSS6*; *PLG*; and *MMP10*
Positive regulation of ERK1 and ERK2 cascade (GO:0070374)	4.63 × 10^−4^	5.48	*PDGFRB*; *FGB*; *THPO*; *CHI3L1*; *ACKR3*; *ADRA1D*; and *ROR2*
Glycosylceramide metabolic process (GO:0006677)	5.73 × 10^−4^	87.61	*LCT* and *ST6GALNAC3*
Positive regulation of MAPK cascade (GO:0043410)	6.53 × 10^−4^	4.06	*PDGFRB*; *GHR*; *FGB*; *FLT1*; *THPO*; *CHI3L1*; *ACKR3*; *ADRA1D*; and *ROR2*

## Data Availability

The mRNA sequence data generated from the 10 mock and SARS-CoV-2 infection-challenged (two replicates each) iPSC-derived AT2 lines (*n* = 40) were submitted to the gene expression omnibus (GEO) archive and are available under the accession number GSE250377.

## Supplementary Materials

The following supporting information can be downloaded at: https://www.mdpi.com/article/10.3390/cells13050369/s1, Table S1: Genes that were significantly differentially (DE) expressed between mock and SARS-CoV-2 infection-challenged AT2s.; Table S2: List of genes whose pre-SARS-CoV-2 infection (mock) expression was significantly correlated with post-infection cellular viral load in AT2s.; Figure S1: Expression heat map of genes whose pre-SARS-CoV-2 infection-challenge expression was significantly correlated with post-SARS-CoV-2 infection viral load.
