# Supplementary material for: Pre-Infection Innate Immunity Attenuates SARS-CoV-2 Infection and Viral Load in iPSC-Derived Alveolar Epithelial Type 2 Cells
Source: Cells. 2024 Feb 21;13(5):369. doi: 10.3390/cells13050369 (PMC10931100; doi:10.3390/cells13050369)
Supplement: Supplementary file 1 [file cells-13-00369-s001.zip › Figure S1.pdf]

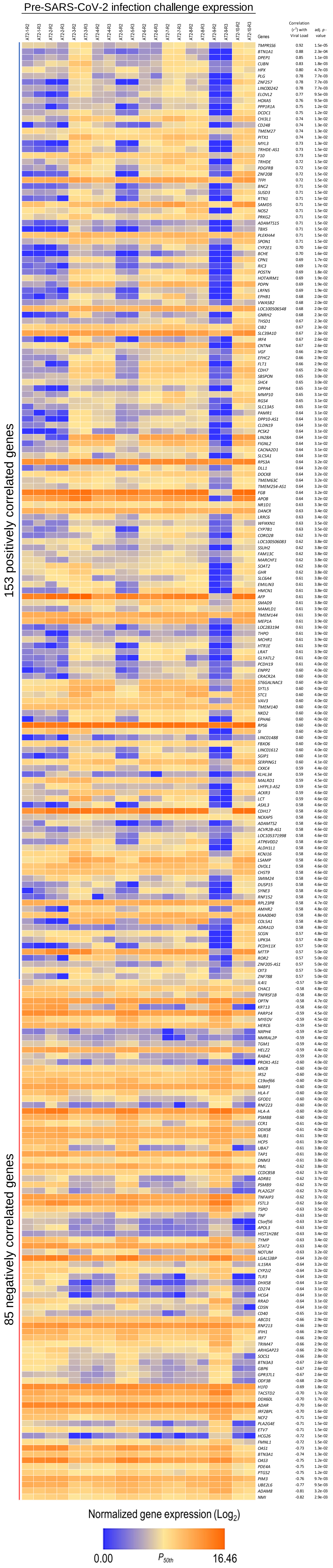

Figure S1. Expression heat map of genes whose pre-SARS-CoV-2 infection challenge expression was significantly correlated with post-SARS-Cov-2 infection viral load.
